# Vam3, a Compound Derived from *Vitis amurensis* Rupr., Attenuated Colitis-Related Tumorigenesis by Inhibiting NF-κB Signaling Pathway

**DOI:** 10.3389/fphar.2016.00311

**Published:** 2016-09-13

**Authors:** Lingling Xuan, Rentao Jiang, Zhiyuan Wu, Honggan Yi, Chunsuo Yao, Qi Hou, Chunfeng Qu

**Affiliations:** ^1^Department of Immunology, Cancer Institute & Hospital, Chinese Academy of Medical Sciences and Peking Union Medical CollegeBeijing, China; ^2^State Key Laboratory of Bioactive Substances and Functions of Natural Medicines, Institute of Materia Medica, Chinese Academy of Medical Sciences and Peking Union Medical CollegeBeijing, China; ^3^State Key Laboratory of Molecular Oncology, National Cancer Center/Cancer Hospital, Chinese Academy of Medical Sciences and Peking Union Medical CollegeBeijing, China

**Keywords:** natural compounds, colitis-related colon cancer, inflammation, mast cells, CD11b^+^Gr1^+^ cells, NF-κB

## Abstract

**Background:** Chronic inflammation is one of the important mediators of colitis-related colon cancer (CRC). Abundant mast cells (MCs) were observed in the tumor microenvironment and mediators released upon MC activation play an important role in the process of chronic inflammation. Previously, we found that activation of intestine mucosal MCs recruited and modulated the inflammatory CD11b^+^Gr1^+^ cells to promote the CRC development. In the current study we investigated the effects of Vam3, a resveratrol dimer with potent anti-inflammatory effects, on CRC development.

**Methods:** RBL-2H3 cells, a basophilic leukemia cell line, were pretreated with 2.5 or 5 µM Vam3 and then stimulated with dinitrophenol-conjugated bovine serum albumin (DNP-BSA) plus lipopolysaccharide (LPS). The MC degranulation was determined by measuring β-hexosaminidase release. Generation of TNF-α and IL-6 in RBL-2H3 cells or in peritoneal macrophages was determined by ELISA and real-time qPCR. NF-κB p65 and phospho-NF-κB p65 expression was determined by Western blotting. NF-κB activity in RAW264.7 cells was determined by luciferase reporter assay. CRC was induced in C57BL/6 mice by intraperitoneal injection of azoxymethane (AOM), followed by oral exposure to dextran sodium sulfate (DSS). Vam3 at 50 mg/kg, or disodium cromoglycate (DSCG, MC stabilizer) at 100 mg/kg, or vehicle were administrated to the mice 4 weeks after DSS withdrawal. Levels of TNF-α, IL-6, and mouse MC protease-1 were determined by ELISA. Infiltration of CD11b^+^Gr1^+^ cells was determined by flow cytometry analysis. One-way ANOVA was used to compare difference between groups.

**Results:** Pretreatment with Vam3 significantly inhibited RBL-2H3 cell degranulation and inflammatory cytokine production from RBL-2H3 cells and from peritoneal macrophages. After Vam3 treatment, NF-κB activity in RAW264.7 cells, and expressions of phospho-NF-κB p65 in RBL-2H3 cells and in peritoneal macrophages were significantly down-regulated. In the AOM plus DSS-induced CRC murine model, the Vam3 and DSCG-treated mice had less tumor numbers than those treated with vehicle. Expression of phospho-NF-κB p65, production of inflammatory cytokines, and infiltration of MCs and CD11b^+^Gr1^+^ cells were attenuated in the Vam3-treated mice.

**Conclusion:** Vam3 treatment could attenuate the CRC development. This effect may be due to its inhibition on NF-κB signaling pathway in MCs and macrophages of the inflamed intestines.

## Introduction

Intrinsic genetic lesion is critical in tumor formation, nevertheless, the importance of chronic inflammation in cancer development has been highlighted recently (Thompson et al., 2015; Romano et al., 2016). Epidemiological and clinical evidences have pointed out that chronic inflammation is one of the most important players in colorectal cancer development (Kraus and Arber, 2009). It was reported that the cumulative incidence of colon cancer in patients with chronic inflammatory bowel disease (IBD) increased to 2, 8, and 20% at 10, 20, and 30 years of disease (Eaden et al., 2001). Anti-inflammatory drugs, such as aspirin, celecoxib, and ibuprofen, have been shown to reduce the incidence of cancer when used as prophylactics, particularly in colon cancer (Harris et al., 2005; Friis et al., 2015). Moreover, the active ingredients of traditional Chinese medicine have gained increasing attention to their applications in the prevention of inflammation-associated cancer risk. Glycyrrhizic acid, a natural and major pentacyclic triterpenoid glycoside of licorice roots extracts, inhibits the development of colon precancerous lesion via regulating the inflammation, hyperproliferation, angiogenesis and apoptosis in the colon of Wistar rats (Khan et al., 2013; Huang et al., 2016). Thus, agents that attenuate intestinal inflammation might be helpful for the prevention of colitis-related colon cancer (CRC).

As the important sentinel cells to the inflammatory stimuli, the role of mast cells (MCs) in promoting the CRC development has been identified in recent years (Gounaris et al., 2007; Khazaie et al., 2011; Tanaka and Ishikawa, 2013; Chen et al., 2015). Based on their morphology, contents of protease, chemistry, and location, MCs are generally divided into two distinct types: mucosal MCs and connective tissue MCs (Gurish and Austen, 2012). In the intestine, mucosal MCs preferentially reside in the epithelium and the lamina propria and express the chymases mouse MC protease (mMCP)-1 and mMCP-2, whereas connective tissue MCs reside in the submucosa and express the chymases mMCP-4 and mMCP-5, and the tryptases mMCP-6, mMCP-7, and carboxypeptidase A (CPA) (Kalesnikoff and Galli, 2008; Khazaie et al., 2011). Mucosal MCs appear to dominate at the site of newly developing polyps, while connective tissue MCs are more prevalent in the stroma at later stages of invasive tumor (Maltby et al., 2009). Gounaris et al. (2007) showed that MCs were the causative agents in polyp formation, the initiating step of colon cancer. Colombo and Pucillo and their colleges reported that MCs were able to counteract the Treg inhibition over effector T cells to play a key role in the inflammation development (Piconese et al., 2009). Furthermore, they demonstrated that MCs boost the activity of myeloid-derived suppressor cells (MDSCs) and contribute to the development of tumor-favoring microenvironment (Danelli et al., 2015a). Previously, we found that activation of intestine mucosal MCs recruited and modulated the inflammatory MDSCs to promote the CRC development (Xu et al., 2015). As their importance as an early source of the inflammatory mediators, MCs, especially the mucosal MCs might be the potential therapeutic targets for preventing CRC development.

Lipopolysaccharide (LPS)/Toll-like receptor-4 (TLR4)/NF-κB pathway plays a crucial role in the development of CRC (Tye and Jenkins, 2013). TLR4 is expressed in many cells, including colonic epithelial cells and the underlying lamina propria immune cells, such as macrophages (Abreu, 2010). Using a colitis-associated cancer model, Fukata et al. (2009) showed that deletion of TLR4 in intestinal epithelial cells could attenuate formation of inflammation-associated tumors. However, mice with TLR4 expression only in the myeloid cells still develop tumors. TLR4 signal from myeloid cells also contribute to some extent to the development of colitis-associated neoplasia (Fukata et al., 2009). TLR4/MyD88 signaling in myeloid cells has been shown to support spontaneous colon cancer development (Grivennikov et al., 2012). Moreover, Greten et al. (2004) showed that deleting IκB kinase (IKK) in myeloid cells resulted in a significant decrease in tumor size. Therefore, it is most likely that LPS/TLR4/NF-κB signaling in both non-immune (epithelial) and immune (myeloid) cell types contribute to the development of colon cancer. Targetting the LPS/TLR4/NF-κB signaling pathway with inhibitors may hold promises in treating colon cancer.

Natural compounds derived from herbal medicine have been reported to have a wide range of biological activities (Newman and Cragg, 2012; Ding et al., 2014). Resveratrol, a polyphenolic compound found in grapes and wine, was reported to possess several beneficial biological effects, including anti-oxidant and anti-inflammatory effects (Yang et al., 2015; Conti et al., 2016; Diaz-Gerevini et al., 2016). Several studies suggest that the chemopreventive effects of resveratrol are partially due to epigenetic regulation and post-transcriptional modifications on the targeted tumor cells (Ross et al., 2008; Saud et al., 2014; Kumar et al., 2015). Recently, studies showed that resveratrol could prevent tumorigenesis in mouse model of Kras activated sporadic colorectal cancer by suppressing oncogenic Kras expression (Saud et al., 2014). Vam3 is a resveratrol dimer derived from *Vitis amurensis* Rupr., which grows in northeastern and central China. Previously we found that Vam3 possessed potent anti-inflammatory and anti-oxidant effects (Shi et al., 2012; Xuan et al., 2014) and was able to inhibit airway inflammation in some animal models (Li et al., 2006; Yang et al., 2010). Vam3 could inhibit macrophages and MCs-mediated inflammatory response (Li et al., 2006; Cao et al., 2014). In the present study, we reported that Vam3 could inhibit RBL-2H3 cell degranulation and decrease cytokine production in RBL-2H3 cells and in peritoneal macrophages. Pretreatment with Vam3 inhibited CRC development in an azoxymethane (AOM) plus dextran sodium sulfate (DSS)-induced CRC murine model. MC infiltration and degranulation, cytokine release and CD11b^+^Gr1^+^ infiltration were attenuated by Vam3 treatment.

## Materials and Methods

### Materials

Compound Vam3, synthesized from resveratrol, was provided by the Institute of Materia Medica, Chinese Academy of Medical Sciences (Beijing, China; Huang et al., 1999). The purity of Vam3 was above 98% as determined by ^1^H-NMR spectra. Rat basophilic leukemia cell lines (RBL-2H3) and murine macrophage cell lines (RAW264.7) were obtained from the American Type Culture Collection (ATCC, Rockville, MD, USA). LPS (serotype O127:B8), AOM, disodium cromoglycate (DSCG), dinitrophenol (DNP)-specific IgE, and DNP-conjugated bovine serum albumin (DNP-BSA) were purchased from Sigma–Aldrich (St. Louis, MO, USA). DSS (MW = 36,000–50,000) was purchased from MP Biomedicals, LLC (Solon, OH, USA). FITC-conjugated anti-mouse CD45 antibodies, PE.Cy7-conjugated anti-mouse CD11b antibodies, and APC-conjugated anti-mouse Gr1 antibodies were purchased from eBioscience (San Diego, CA, USA). Recombinant mMCP-1 was purchased from Sino Biological Inc. (Beijing, China). Anti-CD117 antibody was purchased from Dako (Carpinteria, CA, USA). FuGENE HD Transfection Reagent was purchased from Promega (Madison, WI, USA). Anti-phospho-NF-κB p65 (Ser536) antibodies, anti-NF-κB p65 antibodies, and anti-β-actin antibodies were purchased from Cell Signaling Technology (Beverly, MA, USA).

### Isolation of Peritoneal Macrophages

Primary peritoneal macrophages are used for various *in vitro* studies. However, the yield is typically only ∼0.5–1 × 10^6^ macrophages per mouse. Injecting thioglycollate into peritoneum can increase the yield and purity of macrophages (Zhang et al., 2008).

Peritoneal macrophages were isolated according to the reference (Zhang et al., 2008). Briefly, the male C57BL/6 mice received 1.5 mL of 3% thioglycollate medium via intraperitoneal injection. Four days later, 5 mL ice-cold PBS were injected into the peritoneal cavity. The abdominal cavity was massaged to dislodge loosely adherent peritoneal cells, and the lavage was obtained by recovering of the injected PBS. The procedure was repeated twice for each mouse. The peritoneal lavage fluid was centrifuged at 500 × *g* for 5 min. The cell pellet was washed with ice-cold RPMI-1640 medium containing 1% fetal bovine serum (FBS). The cells were then cultured at 37°C in an air atmosphere containing 5% CO_2_ for 2 h. The non-adherent cells were removed by washing with warmed PBS.

### Cell Viability Assay

RBL-2H3 and RAW264.7 cells were grown in Dulbecco’s Modified Eagle Medium (DMEM) supplemented with 10% FBS_._ Peritoneal macrophages isolated as described above were grown in RPMI-1640 medium containing 10% FBS. Cell viability was assessed using the MTT assay. RBL-2H3, peritoneal macrophages, and RAW264.7 were plated into 96-well plates at a density of 1 × 10^4^ cells per well. Following overnight culture, the cells were then treated with Vam3 (0, 1, 2.5, 5, or 10 μM) for 12, 24, 36, or 48 h. The same volume of DMSO was added to control wells. The cells were subsequently incubated in fresh medium containing MTT solution (0.5 mg/mL) for 4 h at 37°C. The culture medium was removed, and the formazan dye was dissolved in DMSO. The optical densities (OD) at 570 nm were measured with a microplate reader. Relative cell viability was expressed as percentage of controls that were cultured in the absence of Vam3.

### β-Hexosaminidase (β-HEX) Release Assay

RBL-2H3 cells, a rat basophilic leukemia cell line that was widely used in MC-associated studies (Passante and Frankish, 2009), were plated into 96-well plates at a density of 1 × 10^4^ cells per well and sensitized with 0.1 μg/mL DNP-specific IgE for 24 h. The cells were then treated with Vam3 (0, 2.5, or 5 μM) for 2 h followed by adding 1 μg/mL LPS for 48 h. The same volume of DMSO was added to control wells. To test degranulation, the cells were stimulated with 500 ng/mL DNP-BSA for 30 min. Fifty microliters of cell culture supernatant or cell lysates (lysed with 0.5% Triton X-100 to measure residual cell-associated β-HEX) were mixed with an equal volume of substrate solution (2 mM *p*-nitrophenyl-*N*-acetyl-β-_D_-glucosaminide dissolved in 0.1 M sodium citrate buffer, pH 4.5) and incubated for 1 h at 37°C. The enzyme reaction was terminated by adding 200 μL of stop solution (0.1 M Na_2_CO_3_/NaHCO_3_, pH 10.0), and the absorbance was measured at 405 nm with a microplate reader. Release of β-HEX was calculated as a percentage of β-HEX release in the culture medium to the whole β-HEX release in the culture medium and in the cell lysate.

### Quantification of TNF-α and IL-6 in RBL-2H3 Cells and in Peritoneal Macrophages

RBL-2H3 cells (3 × 10^4^/well) and peritoneal macrophages (5 × 10^5^ cells/well) were pretreated with Vam3 (0, 2.5, or 5 μM) for 2 h followed by stimulating with 1 μg/mL LPS for 6 h (for RNA analysis) or for 24 h (for protein analysis). The same volume of DMSO was added to control wells. Total RNA was extracted using TRIzol. First-strand cDNA was synthesized using Prime Script RT Reagent kit (Takara Bio, Otsu, Japan). Real-time qPCR was performed using SYBR Premix Ex Taq (Takara Bio, Otsu, Japan). Primers used for real-time qPCR (**Table 1**) was purchased from SinoGenoMax (Beijing, China). The relative mRNA levels of TNF-α and IL-6 were determined with β-actin or GAPDH as the control and represented as 2^-ΔΔCt^. The cell culture supernatant TNF-α and IL-6 levels were determined using ELISA kits (eBioscience, San Diego, CA, USA).

**Table 1:** List of Primers Used for Real-Time qPCR.

Genes	Forward primer (5′–3′)	Reverse primer (5′–3′)
Murine TNF-α	TGTCCCTTTCACTCAC TGGC	CATCTTTTGGGGGAGT GCCT
Murine IL-6	GGGACTGATGCTGGTG ACAA	TCCACGATTTCCCAGAG AACA
Murine GAPDH	CTCCCACTCTTCCACC TTCG	TAGGGCCTCTCTTGC TCAGT
Rat TNF-α	CGGGCTCAGAATTTCC AACA	CGCAATCCAGGCCACT ACTT
Rat β-actin	GGAGATTACTGCCCCTG GCTCCTA	GACTCATCGTACTCCTGC TTGCTG


### NF-κB Luciferase Reporter Assay

Murine macrophage RAW264.7 cells were plated in 48-well plates at 1 × 10^5^ cells/well and transfected with NF-κB luciferase plasmid using FuGENE HD Transfection Reagent. Twenty-four hours later, the cells were pretreated with Vam3 (0, 2.5, or 5 μM) for 2 h and then stimulated with 1 μg/mL LPS for 24 h. The same volume of DMSO was added to control wells. The cells were lysed with 1x passive lysis buffer. Luciferase activity was measured using the luciferase assay kit (Promega, Madison, WI, USA).

### Quantification of NF-κB Subunit Expression

RBL-2H3 cells and peritoneal macrophages were pretreated with Vam3 (0, 2.5, or 5 μM) for 2 h followed by stimulating with 1 μg/mL LPS for 12 h. The same volume of DMSO was added to control wells. Total protein was extracted using protein extraction kit (Tiangen Biotech, Beijing, China). NF-κB p65 and phospho-NF-κB p65 expression was examined by Western blotting. The density of each band was quantified by QuantiScan Version 11 (Biosoft, Cambridge, UK). Densities of NF-κB p65 and phospho-NF-κB p65 bands were normalized against β-actin.

### Induction of CRC and Treatment with Vam3

Colitis-related colon cancer was induced as previously report (Okayasu et al., 1996). Male C57BL/6, 8–10 weeks, were purchased from the Institute of Laboratory Animal Sciences, Chinese Academy of Medical Sciences (CAMS, Beijing, China). 12.5 mg AOM was dissolved in 5 mL normal saline and injected intraperitoneally at 12.5 mg/kg body weight (5 μL/g body weight). One week later, the mice were exposed orally to 2.5% DSS (MW = 36,000–50,000) for 5 days. Then fresh water was replaced without further treatment. Four weeks after DSS withdrawal, the mice were randomly divided into three groups. One group of the mice (*n* = 9) received DSCG (Oka et al., 2012; Xu et al., 2015) daily at 100 mg/kg of body weight, one group (*n* = 9) received Vam3 daily at 50 mg/kg of body weight, and one group (*n* = 9) were treated with vehicle control (0.1% Tween 80 in distilled water). All the reagents were administrated by gavage for 2 weeks. Mice were sacrificed by CO_2_ asphyxiation 8 weeks after completing the treatment and the colon tissues were collected. Each colon was opened longitudinally and any fecal contents were cleared out with ice-cold saline. The colon weight was measured, and the numbers of polyps were macroscopically assessed individually. The colons were then fixed in 10% formaldehyde and analyzed after staining with hematoxylin and eosin. MCs were stained as reported using toluidine blue (Tanaka et al., 2003). CD117 staining was conducted by using immunohistochemistry as described procedures (Xuan et al., 2014). NF-κB p65 and phospho-NF-κB p65 expression in colon tissues was examined by Western blotting as described above.

### Quantification of mMCP-1, TNF-α, and IL-6 in Serum or in Colon Tissues

To quantify the mMCP-1, TNF-α, and IL-6 in colon tissues, interstitial liquid was prepared as reported (Baronzio et al., 2012). Every 100 mg tissue sample was cut into small pieces in 400 μL ice-cold normal saline and incubated on ice for 5 min. The mMCP-1 concentrations in interstitial liquid and in serum, and the TNF-α and IL-6 concentrations in interstitial liquid were measured using ELISA kits (eBioscience, San Diego, CA, USA) according to the manufacturer’s instructions.

### Flow Cytometry Analysis (FACS) of the Infiltrated Cells in Colon Tissues

Intraepithelial lymphocytes (IEL) and Lamina propria lymphocytes (LPL) were prepared as previously described (Davies and Parrott, 1981). Isolated cells were stained with FITC-conjugated anti-mouse CD45, PE.Cy7-conjugated anti-mouse CD11b, and APC-conjugated anti-mouse Gr1 antibodies. Data were acquired in LSR-II (BD Biosciences, Mountain View, CA, USA) and analyzed by using FlowJo software (Tree Star, Ashland, OR, USA). The analysis was based on the gating of CD45-positive cells.

### Investigation of Vam3 on CD11b^+^Gr1^+^ Cell Recruitment

Male C57BL/6 mice, 8–10 weeks, were randomly divided into three groups (*n* = 3): the naive control group was pretreated with the vehicle control (0.1% Tween 80 in distilled water) and intraperitoneally injected with 0.5 mL DMEM medium; the mMCP-1 group was pretreated with the vehicle control and intraperitoneally injected with 10 ng recombinant mMCP-1 diluted into 0.5 mL DMEM medium; the Vam3 group was pretreated with 50 mg/kg Vam3 and intraperitoneally injected with 10 ng recombinant mMCP-1 diluted into 0.5 mL DMEM medium. Cells in peritoneal cavity were collected and counted 22 h after the mMCP-1 injection. Isolated cells were stained with FITC-conjugated anti-mouse CD45, PE.Cy7-conjugated anti-mouse CD11b, and APC-conjugated anti-mouse Gr1 antibodies for FACS. All procedures involving mice (see Isolation of Peritoneal Macrophages, Induction of CRC and Treatment with Vam3, and Investigation of Vam3 on CD11b^+^Gr1^+^ Cell Recruitment) were approved by the Institutional Animal Care and Use Committee at the Cancer hospital, Chinese Academy of Medical Sciences.

### Statistical Analysis

All statistical analysis were performed using the GraphPad Prism5 program. The statistical analysis was performed using one-way ANOVA to determine difference between the groups. Results were expressed as the means ± SD. A *p*-value of 0.05 or less was considered statistically significant.

## Results

### Vam3 Inhibited the Mast Cell Degranulation and TNF-α Production

**FIGURE 1:**
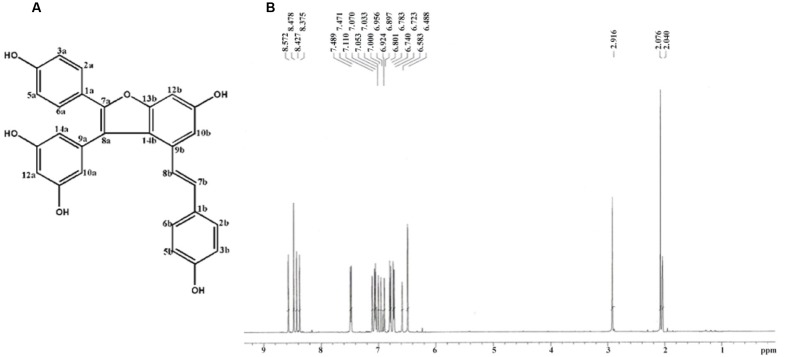
**(A)** Chemical structure of compound Vam3. **(B)** Typical ^1^H-NMR spectra of compound Vam3.

**FIGURE 2:**
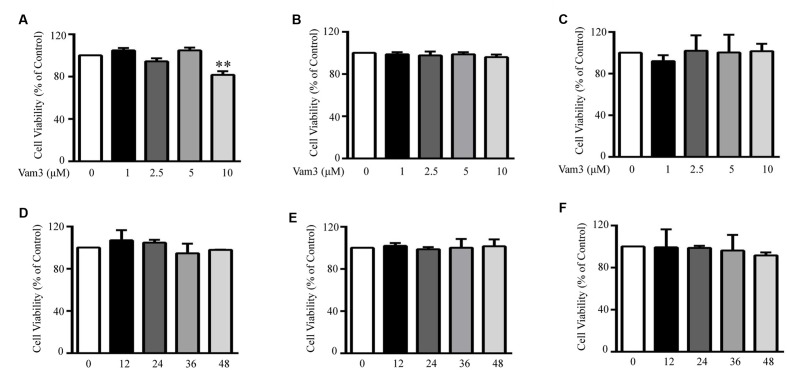
**Effects of Vam3 on the viability of RBL-2H3, peritoneal macrophages and RAW264.7 cells.** RBL-2H3 **(A)**, peritoneal macrophages **(B)**, and RAW264.7 **(C)** plated on 96-well plates were treated with Vam3 (0, 1, 2.5, 5, or 10 µM) for 24 h. RBL-2H3 **(D)**, peritoneal macrophages **(E)**, and RAW264.7 **(F)** were treated with Vam3 (0 or 5 µM) for 0–48 h. Cell viability was assessed using the MTT assay and expressed as percentage of controls that were cultured in the absence of Vam3. ^∗∗^*p* < 0.01 compared with the cells cultured in the absence of Vam3.

The chemical structure and ^1^H-NMR spectra of Vam3 was provided in **Figures 1A,B**, respectively. To test the dose-effect of Vam3 on MC degranulation and cytokine production, 1, 2.5, 5, or 10 μM Vam3 was added to the cell culture medium. The concentration of 1 μM Vam3 showed little effect (data not shown). Therefore, we chose at least 2.5 μM Vam3 for the following work. The cell viability of RBL-2H3, fresh isolated peritoneal macrophages, and RAW264.7 cells was not affected when all these cells were treated with Vam3 (1, 2.5, or 5 μM) for 24 h (**Figures 2A–C**; **Supplementary Figure S1**). No significant cytotoxicity was observed after incubation with 5 μM Vam3 for up to 48 h (**Figures 2D–F**; **Supplementary Figure S1**). However, 10 μM Vam3 reduced the cell viability of RBL-2H3 cells to 81.71 ± 3.28% of control (*p* < 0.01; **Figure 2A**). Therefore, 2.5 and 5 μM were selected for the present study.

**FIGURE 3:**
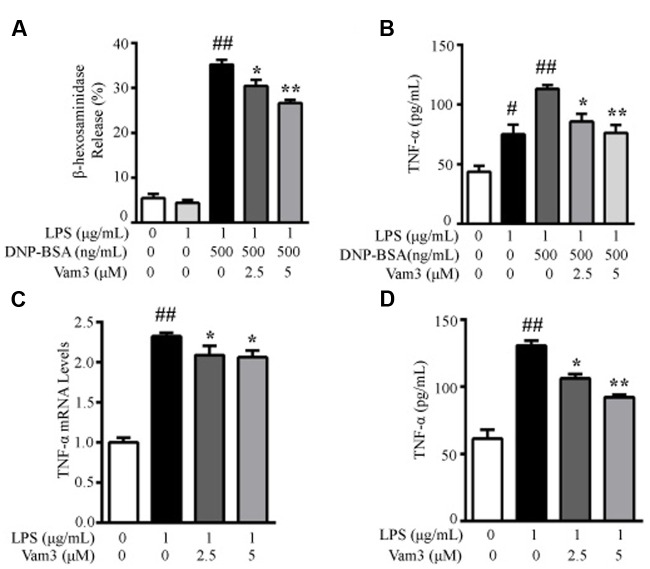
**Effects of Vam3 on MC degranulation and on inflammatory cytokine production.** **(A,B)** RBL-2H3 cells were sensitized with DNP-specific IgE for 24 h and then treated with Vam3 (0, 2.5, or 5 μM) for 2 h followed by adding 1 μg/mL LPS for 48 h. To test degranulation, the cells were stimulated with 500 ng/mL DNP-BSA for 30 min. β-HEX release was measured **(A)** and TNF-α production was quantified by ELISA **(B)**. **(C,D)** RBL-2H3 cells were pretreated with Vam3 (0, 2.5, or 5 μM) for 2 h followed by adding 1 μg/mL LPS for 6 h **(C)** or for 24 h **(D)**. The production of TNF-α was determined by real-time qPCR **(C)** and ELISA **(D)**. ^#^*p* < 0.05, ^##^*p* < 0.01 compared with respective control; ^∗^*p* < 0.05, ^∗∗^*p* < 0.01 compared with the DNP-BSA plus LPS-stimulated group **(A,B)** or the LPS-stimulated group **(C,D)**.

RBL-2H3 cells have been used as a model of intestinal mucosal MCs (Lee et al., 2010; Thrasher et al., 2013). The cells display properties of the intestinal mucosal MCs and share similarities in granule biochemistry with mucosal MCs, such as RMCP-II (Seldin et al., 1985). Therefore, in the present study, we used RBL-2H3 cells as an *in vitro* model of mucosal MCs. Since mucosal MCs are mainly reside within the intestinal mucosa where they are readily exposed to the microbial products, we used DNP-BSA plus LPS-induced degranulation in RBL-2H3 cells as a preliminary screening method to investigate the effects of Vam3 on MC degranulation. Compared with the cells stimulated with DNP-BSA plus LPS, pretreatment with 2.5 and 5 μM Vam3 reduced β-HEX release from an average of 35.21 ± 3.05 to 30.44 ± 3.37% (*p* = 0.023) and 26.61 ± 1.90% (*p* < 0.01), respectively (**Figure 3A**). However, the levels of β-HEX release were still higher in the Vam3-treated groups than baseline (5.46 ± 2.54%).

We then determined the effects of Vam3 on TNF-α release, the main preformed inflammatory cytokine in MCs. The TNF-α production in RBL-2H3 cells stimulated with DNP-BSA plus LPS was 110.37 ± 9.85 pg/mL. When the cells were pretreated with 2.5 and 5 μM Vam3 for 2 h, TNF-α production was reduced to 85.77 ± 11.19 pg/mL (*p* = 0.05) and 76.13 ± 11.55 pg/mL (*p* < 0.01), respectively (**Figure 3B**), but not to baseline level (43.63 ± 8.90 pg/mL). These data suggested that pretreatment with Vam3 could inhibit MC degranulation.

Mast cells could produce pro-inflammatory cytokines, such as TNF-α, in response to LPS without degranulation occurring. So we further investigated the effects of Vam3 on TNF-α production in LPS-stimulated RBL-2H3 cells. Compared with the cells stimulated with LPS alone, pretreatment with 2.5 and 5 μM Vam3 reduced the relative mRNA levels of TNF-α from an average of 2.32 ± 0.04 to 2.09 ± 0.12 (*p* = 0.044) and 2.06 ± 0.08 (*p* = 0.025), respectively (**Figure 3C**), and reduced the protein levels of TNF-α from an average of 130.63 ± 6.49 pg/mL to 106.17 ± 5.79 pg/mL (*p* = 0.022) and 92.20 ± 3.05 pg/mL (*p* < 0.01), respectively (**Figure 3D**). However, the mRNA and protein levels of TNF-α in the Vam3-treated groups were still higher than baseline. These data suggested that pretreatment with Vam3 could inhibit LPS-stimulated TNF-α production in MCs unrelated to degranulation.

### Vam3 Inhibited TNF-α and IL-6 Production from Macrophages

**FIGURE 4:**
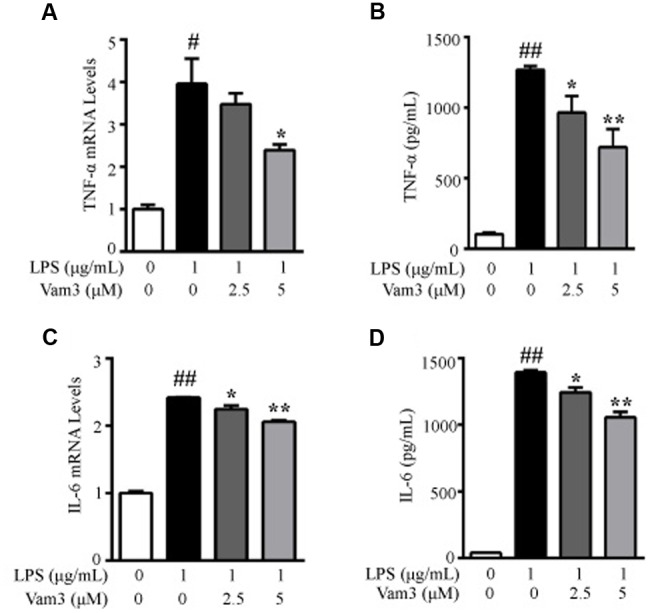
**Effects of Vam3 on inflammatory cytokine production in LPS-stimulated peritoneal macrophages.** **(A,B)** Peritoneal macrophages were pretreated with Vam3 (0, 2.5, or 5 μM) for 2 h followed by adding 1 μg/mL LPS for 6 h **(A)** or for 24 h **(B)**. The production of TNF-α was determined by real-time qPCR **(A)** and ELISA **(B)**. **(C,D)** Similar experimental design to **(A)** and **(B)**. The production of IL-6 was determined by real-time qPCR **(C)** and ELISA **(D)**. ^#^*p* < 0.05, ^##^*p* < 0.01 compared with respective control; ^∗^*p* < 0.05, ^∗∗^*p* < 0.01 compared with the LPS-stimulated group.

Primary peritoneal macrophages are a common source of macrophages for various *in vitro* studies, including stimulation with TLR ligands. One advantage in using peritoneal macrophages is their relative ease of isolation. LPS binds to CD14/TLR4 complex on mouse peritoneal macrophages and stimulates several inflammatory cytokine production, including TNF-α and IL-6 (Akashi et al., 2000). At the site of intestinal inflammation, some intestinal macrophages also express relevant levels of TLR4 and CD14. They produce larger amounts of pro-inflammatory cytokines, such as TNF-α and IL-6, in response to commensal bacteria (Kamada et al., 2008; Yoshioka et al., 2009). Moreover, peritoneal macrophages has been used in colitis and CRC-related studies (Zhao et al., 2014; Yang et al., 2016). Lamina propria macrophages are constantly exposed to microbial products and are a major source of pro-inflammatory cytokines, such as TNF-α and IL-6, in the tumor microenvironment (Terzic et al., 2010). So we further investigated the effects of Vam3 on TNF-α and IL-6 production in LPS-stimulated peritoneal macrophages. Compared with the cells stimulated with LPS alone, pretreatment with 5 μM Vam3 reduced the relative mRNA levels of TNF-α from an average of 3.96 ± 0.59 to 2.40 ± 0.13 (*p* < 0.01; **Figure 4A**). Pretreatment with 2.5 and 5 μM Vam3 reduced the protein levels of TNF-α from an average of 1268.02 ± 25.82 pg/mL to 964.01 ± 119.39 pg/mL (*p* = 0.032) and 718.88 ± 129.13 pg/mL (*p* < 0.01), respectively (**Figure 4B**). Meanwhile, when the cells were pretreated with 2.5 and 5 μM Vam3 for 2 h, the relative mRNA levels of IL-6 decreased from an average of 2.42 ± 0.01 to 2.24 ± 0.05 (*p* = 0.038) and 2.06 ± 0.02 (*p* < 0.01), respectively (**Figure 4C**), and the protein levels of IL-6 decreased from an average of 1393.46 ± 30.55 pg/mL to 1242.89 ± 64.33 pg/mL (*p* = 0.038) and 1056.01 ± 71.28 (*p* < 0.01), respectively (**Figure 4D**). However, the mRNA and protein levels of TNF-α and IL-6 in the Vam3-treated groups were still higher than baseline. These data suggested that pretreatment with Vam3 could inhibit LPS-stimulated cytokine production in macrophages.

### Vam3 Inhibited CRC Development

**FIGURE 5:**
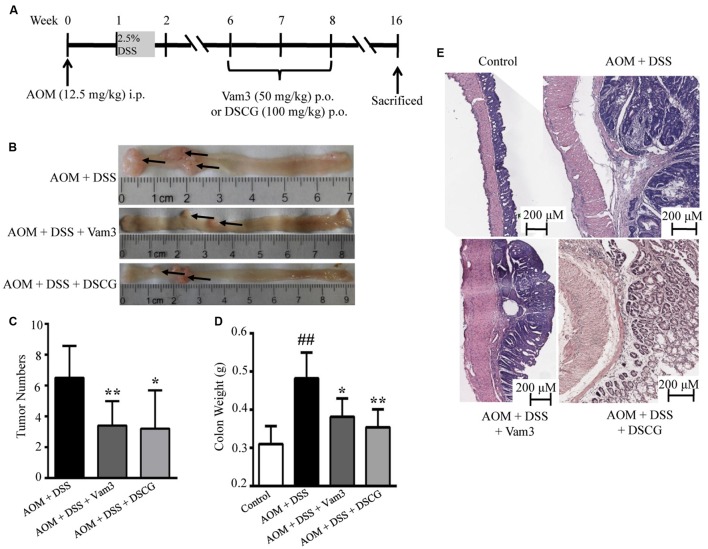
**Effects of Vam3 on CRC development in murine model.** **(A)** Schematic diagram of the experimental design. CRC was induced by AOM (12.5 mg/kg) plus DSS (2.5%) in male C57BL/6 mice. Mice were treated with either vehicle, Vam3 (50 mg/kg) or DSCG (100 mg/kg) by oral gavage for 2 weeks. **(B)** Macroscopic appearance of colon tissues. **(C)** Total tumor numbers. **(D)** Colon weight measurement. **(E)** Representative hematoxylin and eosin sections of colon tissues (100×). *n* = 5–9 per group. ^##^*p* < 0.01 compared with the control group. ^∗^*p* < 0.05, ^∗∗^*p* < 0.01 compared with the AOM plus DSS-induced group.

The CRC murine model was induced by intraperitoneal injection with procarcinogen AOM followed by one cycle of DSS exposure. Four weeks after DSS withdrawal, the mice were treated with Vam3, DSCG, and vehicle for 2 weeks (**Figure 5A**). All of the mice developed colon tumors 14 weeks after DSS withdrawal. However, Vam3 and DSCG treatment reduced total tumor numbers from an average of 6.50 ± 2.07/mice in the vehicle-treated mice to 3.40 ± 1.58/mice (*p* < 0.01) and 3.20 ± 2.49/mice (*p* = 0.018), respectively (**Figures 5B,C**). Treatment with Vam3 and DSCG reduced colon weight from an average of 0.48 ± 0.03 g to 0.38 ± 0.02 g (*p* = 0.050) and 0.35 ± 0.02 g (*p* = 0.010), respectively (**Figure 5D**). The effect of Vam3 was not different from that of DSCG in **Figures 5C,D** (*p* > 0.05). Histologically, the vehicle-treated mice showed more destruction of the colonic epithelium, a thicker muscularis layer and adenocarcinoma development. However, the structure of colonic epithelium was improved in the Vam3 and DSCG-treated mice (**Figure 5E**). These results collectively indicated that Vam3 treatment could inhibit CRC development in the murine model.

### Vam3 Inhibited the Mast Cell Infiltration and Attenuated Cytokine Production *In vivo*

**FIGURE 6:**
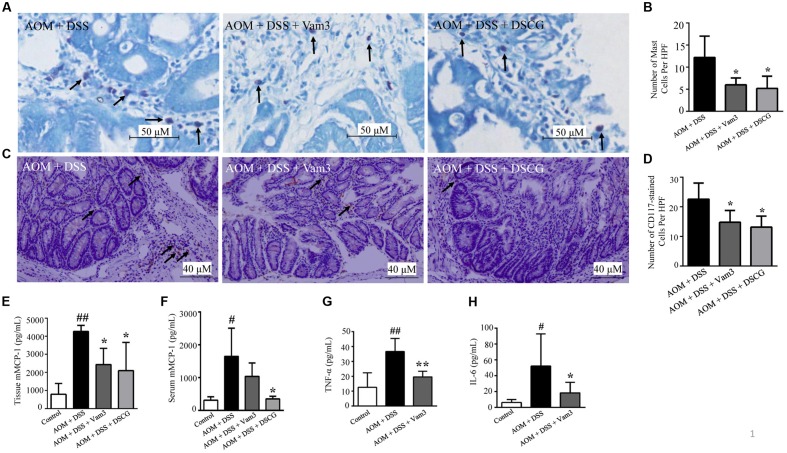
**Effects of Vam3 on MC infiltration and on cytokine production in colon tissues.** **(A)** MCs stained by toluidine blue (400×). **(B)** Numbers of toluidine blue-stained MCs. **(C)** Immunohistochemical staining for CD117 (200×). **(D)** Numbers of CD117-positive cells. **(E,F)** mMCP-1 levels in interstitial fluid **(E)** and in serum **(F)** determined by ELISA. **(G,H)** TNF-α and IL-6 levels in colon tissues determined by ELISA. *n* = 5–9 per group. ^#^*p* < 0.05, ^##^*p* < 0.01 compared with the control group. ^∗^*p* < 0.05, ^∗∗^*p* < 0.01 compared with the AOM plus DSS-induced group.

As Vam3 could inhibit RBL-2H3 cell degranulation and decrease cytokine production in RBL-2H3 cells and macrophages, we hypothesized that the inhibitory effect of Vam3 on CRC development may be due to its inhibition on MC degranulation and cytokine production. As shown in **Figures 6A,B**, the numbers of toluidine blue-stained MCs were reduced in the Vam3 (*p* = 0.037) and DSCG (*p* = 0.018)-treated relative to the vehicle-treated mice. We then stained the colon tissues with antibody against CD117. CD117 is an early marker for MC precursors and expressed throughout their lifetime (Hodges et al., 2012). The numbers of CD117-positive cells were also reduced in the Vam3 (*p* = 0.050) and DSCG (*p* = 0.017)-treated relative to the vehicle-treated mice (**Figures 6C,D**). The effect of Vam3 was not different from that of DSCG in reducing MC infiltration (*p* > 0.05).

Interstitial fluid is the physical and biochemical microenvironment of the tumor cells. Post-translational modifications may occur in interstitial fluid. Studies highlight the importance of quantification of substances in the interstitial fluid (Garvin and Dabrosin, 2003; Iversen and Wiig, 2005). Garvin and Dabrosin (2003) showed that intracellular levels of vascular endothelial growth factor (VEGF) were increased by tamoxifen. However, tamoxifen treatment decreased secretion of VEGF into extracellular phase and decreased angiogenesis *in vivo* (Garvin and Dabrosin, 2003). Therefore, it is important to monitor biomolecules in the compartment where they are biologically active. In the present study, mMCP-1, TNF-α, and IL-6 levels were quantified in interstitial liquid.

The mMCP-1 is predominantly expressed by intestinal mucosal MCs. After activation and degranulation of the mucosal MCs, mMCP-1 is released from mucosal MCs. Treatment with Vam3 and DSCG reduced interstitial liquid mMCP-1 levels from an average of 4266.94 ± 329.48 pg/mL in the vehicle-treated mice to 2433.57 ± 896.16 pg/mL (*p* = 0.050) and 2098.89 ± 1559.16 pg/mL (*p* = 0.016), respectively (**Figure 6E**). The effect of Vam3 was not different from that of DSCG in **Figure 6E** (*p* > 0.05). The mMCP-1 levels in serum did not decrease significantly after Vam3 treatment (**Figure 6F**). However, as mentioned above, it is important to monitor biomolecules in the compartment where they are biologically active, such as interstitial fluid. These data indicated that Vam3 treatment inhibited MC infiltration and activation *in vivo*.

TNF-α and IL-6, expressed in the tumor microenvironment, are important for the development of inflammation-associated intestinal tumorigenesis. Treatment with Vam3 reduced TNF-α levels from an average of 36.65 ± 8.80 pg/mL in the vehicle-treated mice to 19.61 ± 3.71 pg/mL (*p* < 0.01; **Figure 6G**). Meanwhile, the mice treated with Vam3 revealed a lower IL-6 level, averaging 18.37 ± 13.33 pg/mL, than the mice treated with vehicle (52.10 ± 40.60 pg/mL) (*p* = 0.034) (**Figure 6H**). These results collectively indicated that Vam3 treatment could inhibit the MC infiltration and degranulation, and attenuated cytokine production *in vivo.*

### Vam3 Inhibited CD11b^+^Gr1^+^ Cell Infiltration through Suppression of MC Degranulation

**FIGURE 7:**
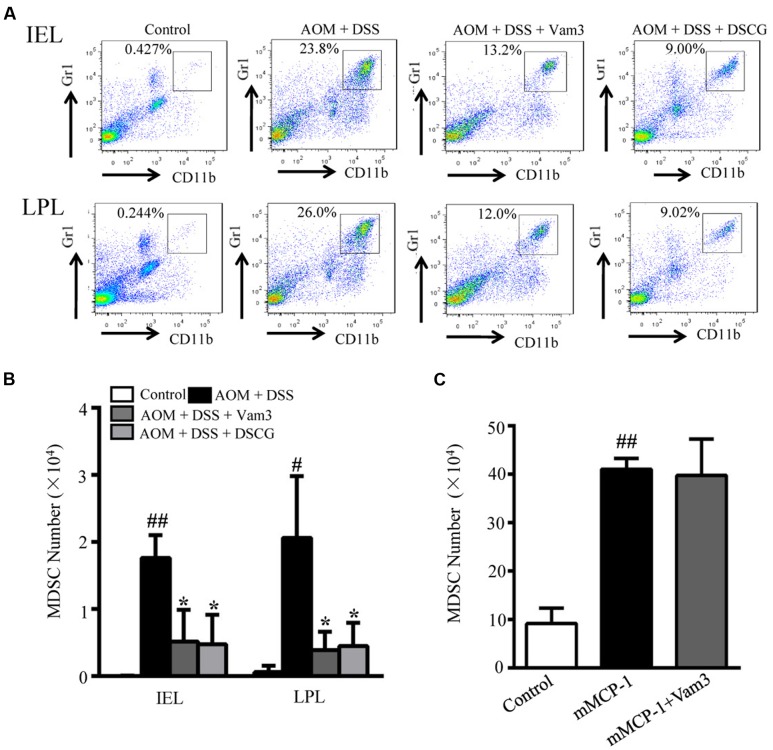
**Effects of Vam3 on CD11b^+^Gr1^+^ cell infiltration.** **(A)** Representative FACS of CD11b^+^Gr1^+^ cells in IEL and LPL of AOM plus DSS-induced CRC murine model. **(B)** The numbers of CD11b^+^Gr1^+^ cells in IEL and LPL of AOM plus DSS-induced CRC murine model. **(C)** The mice were pretreated with Vam3 or vehicle before intraperitoneally injection with recombinant mMCP-1 or DMEM medium as described in section “Materials and Methods.” Twenty-two hours after the mMCP-1 injection, cells infiltrated into the peritoneal cavity were collected using 5 mL of DMEM medium. The numbers of CD11b^+^Gr1^+^ cells in each group were determined by FACS. *n* = 3–9 per group. ^#^*p* < 0.05, ^##^*p* < 0.01 compared with the control group. ^∗^*p* < 0.05 compared with the AOM plus DSS-induced group.

CD11b^+^Gr1^+^ cells were reported to be the immunosuppressive cell population in the tumor microenvironment that favors tumor growth and could suppress T cell activity (Wesolowski et al., 2013). mMCP-1 released from mucosal MCs could induce CD11b^+^Gr1^+^ cell accumulation (Xu et al., 2015). We then determined the effects of Vam3 treatment on CD11b^+^Gr1^+^ cell infiltration by FACS. The percentage and numbers of CD11b^+^Gr1^+^ cells in both IEL and LPL were reduced in the Vam3 and DSCG-treated relative to the vehicle-treated mice (*p* < 0.05 for all; **Figures 7A,B**). The effect of Vam3 was not different from that of DSCG (*p* > 0.05).

To test whether Vam3 may directly inhibit CD11b^+^Gr1^+^ cell infiltration, mice were treated with Vam3 and recombinant mMCP-1 was injected into mouse peritoneal cavity, where no mucosal MCs reside. Consistent with previous study (Xu et al., 2015), the CD11b^+^Gr1^+^ cells in the peritoneal cavity increased significantly in the mice that received mMCP-1 (*p* < 0.01). However, there were no significant differences in CD11b^+^Gr1^+^ cell numbers between the vehicle and Vam3-treated mice that received mMCP-1 (*p* > 0.05; **Figure 7C**). These results indicated that Vam3 treatment did not directly inhibit CD11b^+^Gr1^+^ cell infiltration. It is most likely that Vam3 inhibited CD11b^+^Gr1^+^ cell infiltration through suppression of MC degranulation.

### Vam3 Inhibited NF-κB Activation

**FIGURE 8:**
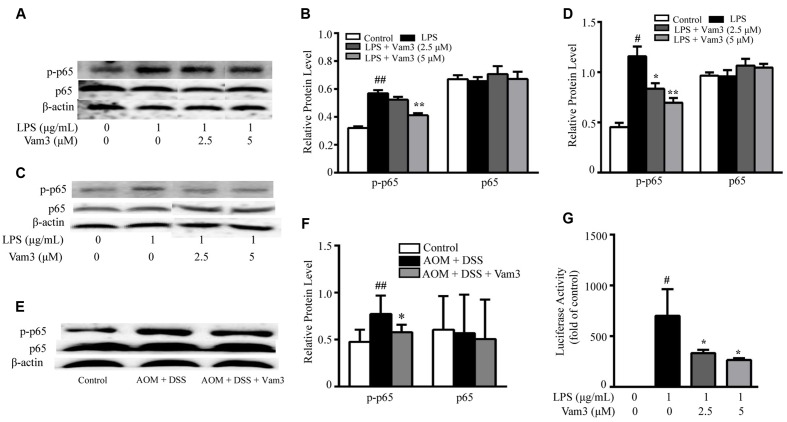
**Effects of Vam3 on NF-κB activation.** **(A,B)** RBL-2H3 cells were pretreated with Vam3 (0, 2.5, or 5 μM) for 2 h followed by adding 1 μg/mL LPS for 12 h. NF-κB p65 and phospho-NF-κB p65 expression was examined by Western blotting **(A)**, and quantification of the NF-κB p65 and phospho-NF-κB p65 expression was performed by densitometric analysis of the blots **(B)**. **(C,D)** Similar experimental design to **(A)** and **(B)** using peritoneal macrophages. **(E,F)** Colon tissues from each group were used for NF-κB p65 and phospho-NF-κB p65 expression measurement using Western blotting (*n* = 9 per group). **(G)** RAW264.7 cells were transiently transfected with NF-κB luciferase plasmids. Transfected cells were pretreated with Vam3 (0, 2.5, or 5 μM) for 2 h followed by adding 1 μg/mL LPS for 24 h. Cells were lysed and the lysates were analyzed by use of a luciferase assay system. ^#^*p* < 0.05, ^##^*p* < 0.01 compared with the control group. ^∗^*p* < 0.05, ^∗∗^*p* < 0.01 compared with the LPS-stimulated group or the AOM plus DSS-induced group.

NF-κB plays a central role in DNP-BSA plus LPS-induced MC degranulation and in LPS-induced cytokine production in MCs and macrophages. In the NF-κB signaling cascade, phosphorylation of p65 at the serine 536 position is required for nuclear translocation (Maguire et al., 2015). Therefore, to determine whether Vam3 exhibited its effect via the NF-κB pathway, expression of NF-κB p65 and phospho-NF-κB p65 were determined in the RBL-2H3 cells, peritoneal macrophages and colon tissues. Compared with the RBL-2H3 cells stimulated with LPS alone, pretreatment with 5 μM Vam3 reduced the relative phospho-NF-κB p65 expression from an average of 0.57 ± 0.02 to 0.41 ± 0.02 (*p* < 0.01; **Figures 8A,B**). In a similar pattern, LPS significantly increased phospho-NF-κB p65 expression in peritoneal macrophages (*p* < 0.01) whereas Vam3 pretreatment at 2.5 μM (*p* = 0.044) and 5 μM (*p* < 0.01) reduced this change (**Figures 8C,D**). Consistent with the *in vitro* studies above, treatment with Vam3 reduced relative phospho-NF-κB p65 expression in colon tissues from an average of 0.77 ± 0.20 to 0.58 ± 0.08 (*p* = 0.025; **Figures 8E,F**; **Supplementary Figure S2**). However, NF-κB p65 levels were not changed in LPS-stimulated RBL-2H3 cells and peritoneal macrophages and in colon tissues.

In addition, we used an NF-κB-dependent luciferase assay to further determine the effect of Vam3 on NF-κB activity in LPS-stimulated RAW264.7 cells. Compared with the cells stimulated with LPS alone, pretreatment with 2.5 and 5 μM Vam3 reduced the relative luciferase activity from 700.41 ± 260.86-fold of the control to 331.59 ± 32.65-fold (*p* = 0.050) and 263.77 ± 19.06-fold (*p* = 0.022) of the control, respectively (**Figure 8G**). All these findings indicated that Vam3 treatment inhibited NF-κB activation.

## Discussion

In this study, we investigated the effects of compound Vam3, a resveratrol dimer with potent anti-inflammatory and anti-oxidant activities (Li et al., 2006; Shi et al., 2012), on colitis-associated colon carcinogenesis. Pretreatment with Vam3 could inhibit DNP-BSA plus LPS-induced RBL-2H3 cell degranulation and decrease cytokine production in LPS-stimulated RBL-2H3 cells and peritoneal macrophages. In the AOM plus DSS-induced CRC murine model, the Vam3-treated mice had less tumor numbers than the vehicle-treated mice. Expression of phospho-NF-κB p65, production of inflammatory cytokines, and infiltration of MCs and CD11b^+^Gr1^+^ cells were attenuated in the intestines of the Vam3-treated mice compared to the vehicle-treated mice. These data demonstrated that Vam3 treatment inhibited CRC development and this effect was likely associated with its inhibition on NF-κB signaling pathway.

Many studies have documented the critical link between inflammation and the development of colon cancer (Kraus and Arber, 2009; Terzic et al., 2010). The microbiota directly and indirectly affects the development and maintenance of inflammation, which is the hallmark of cancer (Kostic et al., 2013; Dzutsev et al., 2015). Human intestine harbors over 100 trillion bacteria, and colon is the most densely populated intestinal region for bacteria (Sears and Garrett, 2014). Involvement of commensal bacteria and its components with strong immunoactivating properties, such as LPS, has been suggested in the histopathology of colon cancer (Tlaskalova-Hogenova et al., 2004; Collins et al., 2011; Sears and Garrett, 2014). Altered epithelial barrier function induces the translocation of luminal antigens, such as LPS, from the gut lumen into the bowel wall, which leads to immune cell activation and cytokine production (Chichlowski and Hale, 2008; Neurath, 2014). LPS has been suggested to have no effect on MC degranulation. However, recent studies have shown that prolonged treatment with LPS resulted in enhancement of MC degranulation following IgE-receptor cross-linking (Saluja et al., 2012; Yang et al., 2012). Intestinal MCs activation through FcεRI may play a role in the pathogenesis of IBD. It was reported that treatment with the anti-IgE antibody could reduce the expression of MC-mediated inflammatory factors in DSS-induced colitis (Kang et al., 2006). However, the mechanisms of MC degranulation in CRC remain largely unclear. IgE/Ag-activated bone marrow-derived MCs (BMMCs) was used by Danelli et al. (2015a) to study MC degranulation on colon tumor development. The results showed that IgE/Ag-activated BMMCs not only had the potential to induce MDSCs recruitment, but also contribute to the acquisition of suppressive functions by MDSCs (Danelli et al., 2015a).

Mucosal MCs predominantly localize to the mucosal epithelium and lamina propria where they are readily exposed to microbial products. RBL-2H3 cells have characteristics of mucosal MCs (Barsumian et al., 1981). Therefore, in this study, we used DNP-BSA plus LPS-induced degranulation in RBL-2H3 cells as a preliminary screening method to investigate the effects of Vam3 on MC degranulation. Activated MCs degranulate and release preformed mediators such as histamine, neutral proteases, proteoglycans, and some cytokines, such as TNF-α. Among the preformed mediators, β-HEX is a marker of MC degranulation (Fukuishi et al., 2014). The present study showed that pretreatment with Vam3 could inhibit DNP-BSA plus LPS-induced β-HEX release in RBL-2H3 cells. TNF-α is a key pro-inflammatory cytokine involved in the development of CRC (Trejo et al., 2001; Kraus and Arber, 2009). MCs are the only cell type capable of storing pre-synthesized TNF-α and have been suggested as an important source for TNF-α in IBD (Gounaris et al., 2007; Kalesnikoff and Galli, 2008). The release of TNF-α in DNP-BSA plus LPS-induced RBL-2H3 cells was attenuated by Vam3 pretreatment. MCs could produce pro-inflammatory cytokines in response to LPS, without degranulation occurring. The release of TNF-α was also attenuated by Vam3 pretreatment in LPS-induced RBL-2H3 cells. Most tumor-promoting cytokines, such as TNF-α and IL-6 are produced by lamina propria macrophages and dendritic cells (DC) during early states of CRC development (Terzic et al., 2010). Vam3 pretreatment significantly inhibited the release of TNF-α and IL-6 in LPS-induced peritoneal macrophages.

Acute toxicity tests in mice showed that the maximal tolerance dose of Vam3 was greater than 4 g/kg body weight. The growth and general behavior of the animals appeared normal and no abnormality was observed in major organs (unpublished data). Previously we found that oral administration of Vam3 at about 50 mg/kg inhibited inflammatory cell recruitment, cytokine production and NF-κB activation in ovalbumin (OVA)-induced asthma murine model (unpublished data), and showed anti-oxidative effect on cigarette smoke-induced damage (Shi et al., 2012). Therefore, a dosage of 50 mg/kg was used in the present study. Vam3 treatment at 50 mg/kg not only reduced MC infiltration and degranulation but also reduced the production of TNF-α and IL-6 in an AOM plus DSS-induced CRC murine model. These results indicated that Vam3 treatment attenuated CRC development probably by inhibiting MC degranulation and cytokine release from MCs and macrophages.

Myeloid-derived suppressor cells are a heterogeneous population of immature myeloid cells at different stages of cell differentiation and defined as CD11b^+^Gr1^+^ in mice. MDSCs are responsible for T cell suppression and the formation of the immunosuppressive tumor microenvironment that favors tumor growth. Recent studies highlight the importance of the bidirectional axis between MDSCs and MCs in creating tumor-inflammatory and tumor-immunosuppressive microenvironments (Danelli et al., 2015b). Our current study found that mMCP-1 released from mucosal MCs induced CD11b^+^Gr1^+^ cell accumulation and further amplified the potential of CD11b^+^Gr1^+^ cells to enhance tumor cell proliferation and inhibit T cell activation (Xu et al., 2015). As Vam3 could attenuate MC infiltration and degranulation, we then determined the effects of Vam3 on MDSC accumulation. As expected, the percentage and numbers of CD11b^+^Gr1^+^ cells decreased in the colon tissues of the Vam3 treatment group. However, Vam3 treatment did not directly inhibit mMCP-1-induced CD11b^+^Gr1^+^ cell infiltration *in vivo*. It is most likely that Vam3 inhibited CD11b^+^Gr1^+^ cell infiltration through suppression of MC degranulation.

Aberrant or constitutive NF-κB activation has been observed in many cancers including CRC. Greten et al. (2004) showed that specific inactivation of the IKK/NF-κB pathway in myeloid cells can attenuate formation of inflammation-associated tumors in an AOM plus DSS-induced CRC murine model. NF-κB activation in premalignant cells induce genes that stimulate cell proliferation and survival. NF-κB also plays an essential role in the production of chemokine and cytokine that sustain tumor-associated inflammation (Grivennikov et al., 2010). LPS recognition by TLR4/MD2/CD14 initiates the intracellular signaling cascade in a MyD88-dependant or MyD88-independent manner. This finally results in the activation of a series of signaling events, such as NF-κB, that potentiate the production of inflammatory mediators. Recent studies showed that activation of the TLR4/Myd88/NF-κB pathway is crucial for LPS-induced regulation of Ca^2+^ mobilization and MC degranulation (Yang et al., 2012). Since NF-κB activation is involved in LPS plus DNP-BSA-induced MC degranulation and LPS-induced cytokine release, we hypothesized that the inhibitory effect of Vam3 on CRC development might be related to NF-κB inhibition. In our study, we found that Vam3 administration inhibited the phosphorylation of NF-κB p65 in colon tissues. Furthermore, pretreatment with Vam3 decreased phospho-NF-κB p65 levels in LPS-stimulated peritoneal macrophages and RBL-2H3 cells, and decreased NF-κB-luciferase activity in LPS-stimulated RAW264.7 cells. These findings suggest that the effects of Vam3 were at least in part mediated by inhibiting the NF-kB pathway. The exact mechanism of how Vam3 inhibits NF-κB activation will require further investigations.

**FIGURE 9:**
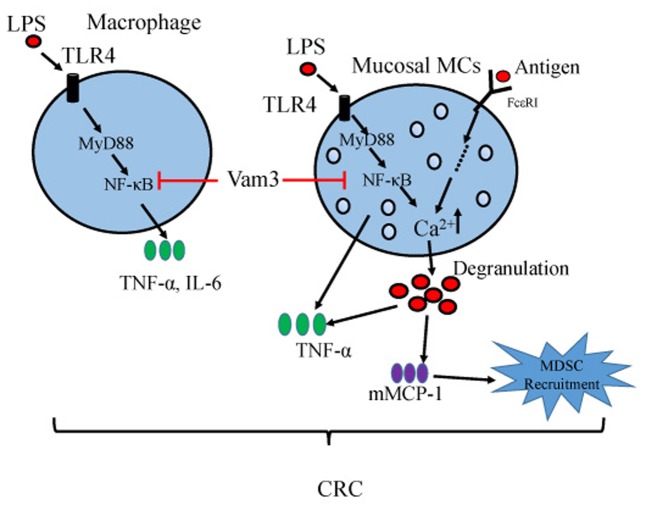
**Proposed model for Vam3 inhibiting colitis-related tumorigenesis**.

In summary, our results showed that administration of Vam3 could attenuate the CRC development in an AOM plus DSS-induced CRC murine model. This effect may be due to its inhibition on NF-κB signaling pathway in MCs and macrophages of the inflamed intestines (**Figure 9**). Vam3 might be a potential prophylactic agent for CRC therapy.

## Author Contributions

LX designed and performed the experiments, analyzed the data, and drafted the manuscript. RJ assisted in *in vitro* experiments. ZW and HY assisted in animal experiments. CY carried out chemical synthesis. QH designed the study and revised the paper. CQ designed the study, drafted and revised the manuscript. All authors read and approved the final manuscript.

## Conflict of Interest Statement

The authors declare that the research was conducted in the absence of any commercial or financial relationships that could be construed as a potential conflict of interest.
