# Supplementary figures and images for: Vam3, a Compound Derived from Vitis amurensis Rupr., Attenuated Colitis-Related Tumorigenesis by Inhibiting NF-κB Signaling Pathway
Source: Front Pharmacol. 2016 Sep 13;7:311. doi: 10.3389/fphar.2016.00311 (PMC5020048; doi:10.3389/fphar.2016.00311)

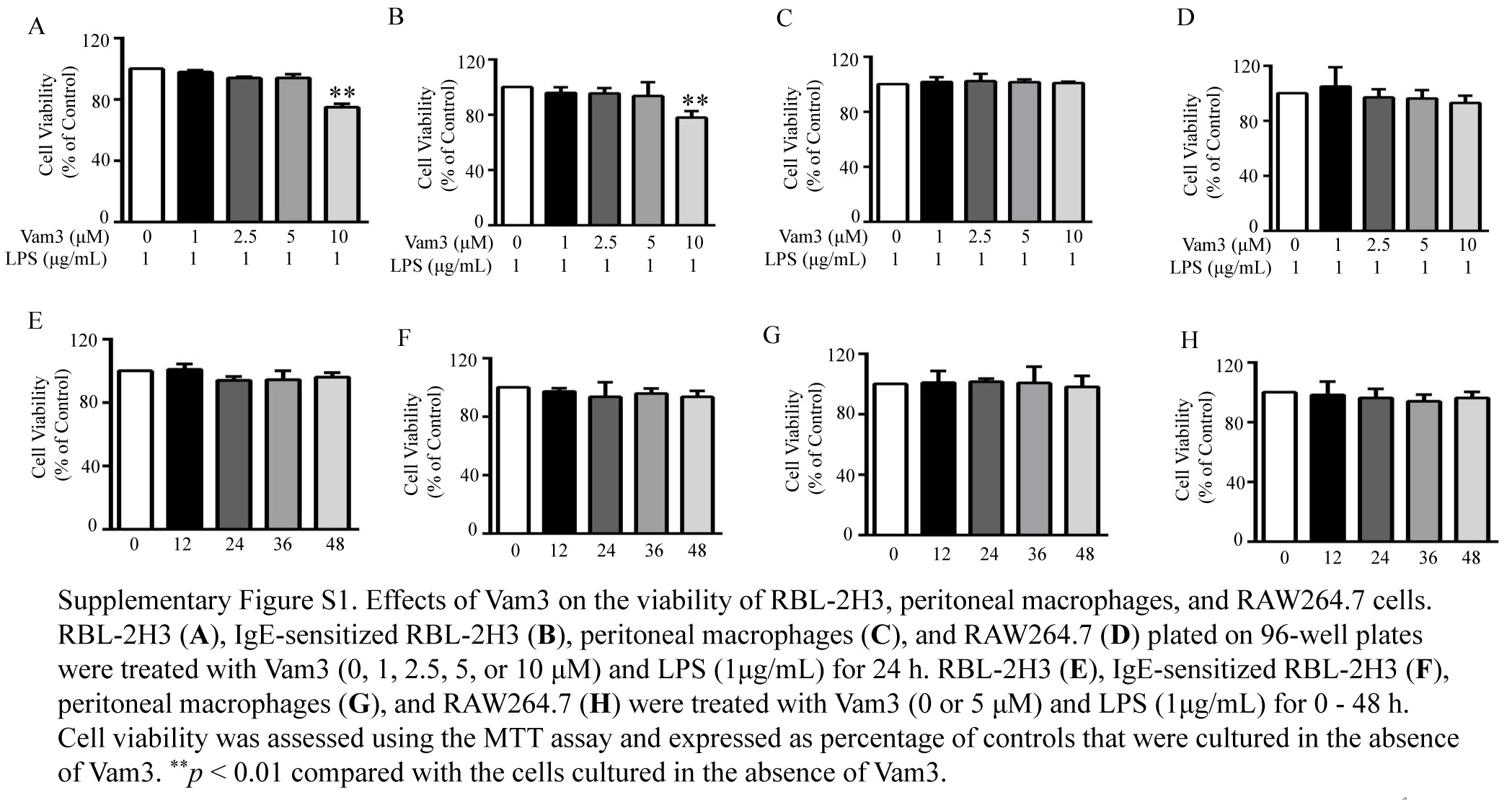

Supplement: Supplementary file 1 [file Image_1.JPEG]

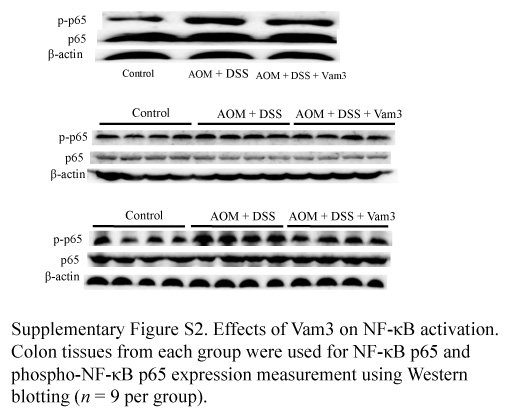

Supplement: Supplementary file 2 [file Image_2.JPEG]
